# Psychological distress among nursing students during the COVID-19 pandemic: a hybrid concept analysis

**DOI:** 10.1186/s40359-025-02562-x

**Published:** 2025-03-08

**Authors:** Masoud Feyzbabaie, Nahid Rajai, Azizeh Alizadeh, Maryam Azizi

**Affiliations:** 1 Cardiovascular Nursing Research Center, Rajaie Cardiovascular Medical and Research Center, Iran University of Medical Sciences, Tehran, IR Iran; 2 Intensive Care Nursing Department, Aja University of Medical Sciences, Tehran, IR Iran; 3 NEZAJA Health Department, Assistance of Mental Health in Khanevadeh Hospital, Tehran, IR Iran; 4 Health in Disaster and Emergencies Department, Faculty of Nursing, Aja University of Medical Sciences, Tehran, IR Iran

**Keywords:** Psychological distress, Nursing students, COVID-19, Hybrid

## Abstract

**Background:**

This study aimed to investigate the concept of psychological distress among nursing students during the COVID-19 pandemic. Understanding its dimensions and characteristics of this phenomenon can enhance preparedness for future pandemics. Psychological distress has emerged as a significant mental health concern during the pandemic, with nursing students experiencing high levels of psychological distress caused by substantial disruptions in their educational environment.

**Method:**

This study employed the Schwartz-Barcott and Kim’s hybrid concept analysis model, integrating a systematic literature review with qualitative research to examine psychological distress among nursing students during the COVID-19 pandemic. The literature review included a comprehensive search across multiple databases, resulting in the identification of 60 relevant articles for data extraction. In the qualitative phase, semi-structured interviews were carried out with nursing students from the Army Nursing Faculty, and the data were analyzed which were analyzed using a directed content analysis approach. The findings from both phases were synthesized to provide a comprehensive definition of psychological distress in nursing students during the pandemic.

**Results:**

Psychological distress among nursing students during the COVID-19 pandemic was analyzed through three key dimensions: antecedents, characteristics, and consequences. Antecedents included factors such as personality traits, demographic factors, social influences, and health-related conditions, with demographics standing out as particularly impactful. The characteristics of distress were categorized into emotional-psychological, cognitive, and physical symptoms, with sleep disturbances being especially prominent. The consequences encompassed both negative outcomes—like academic setbacks, social withdrawal, and physical health problems—and positive outcomes, such as post-traumatic growth, improved coping skills, and professional advancement. The findings offer a thorough understanding of the multifaceted nature of psychological distress and its effects on nursing students.

**Conclusion:**

The findings of this study explore the antecedents, characteristics, and consequences of students’ psychological distress, providing essential insights for health policymakers and educational planners during similar pandemics. This data can inform the development, planning, and implementation of treatment and training systems designed to prevent such conditions in future pandemics. In essence, by identifying and addressing the underlying factors or antecedents of this distress, its occurrence in future pandemics could be effectively reduced.

**Supplementary Information:**

The online version contains supplementary material available at 10.1186/s40359-025-02562-x.

## Introduction

Coronavirus first emerged on December 17, 2019, in Wuhan, China, and on March 11, 2020, the World Health Organization officially declared it a global pandemic [1]. As of August 28, 2023, approximately 7 million deaths worldwide were attributed to COVID-19, with approximately 150,000 deaths reported in Iran [2]. On May 5, 2023, three years later, the World Health Organization announced the end of the public health emergency related to COVID-19, while underscoring the continued presence of the virus despite widespread vaccination, testing, and treatment efforts [3]. The pandemic brought widespread anxiety due to the unknown nature of the disease, underprepared healthcare systems, rapid transmission, severe physical complications, high mortality rates, stigma [4], financial instability, and the disruption of daily life caused by quarantine measures [5]. Repeated waves of infection, each with unique characteristics, created confusion for both healthcare workers and the public, leading to significant psychological strain. For many, symptoms persisted long after recovery, and those affected by these long-term effects, often referred to as “long haulers,” have faced ongoing physical, mental, and emotional challenges, further straining individuals and the healthcare system [6–8].

During the COVID-19 pandemic, psychological distress has emerged as a critical mental health concern [9–12]. It is characterized by an unpleasant state of depression and anxiety with both physical and emotional manifestations, often accompanied by psychosocial and behavioral symptoms [13, 14]. It can be described as an overwhelming or uncomfortable emotional state triggered by life events, stressors, or personal challenges [15]. Psychological distress is a broad term encompassing various psychological conditions, ranging from mild, subclinical symptoms to more severe conditions such as depression, anxiety, stress, or post-traumatic stress disorder [16]. Unlike anxiety and depression, which are specific mental health conditions with defined diagnostic criteria, psychological distress does not always meet the diagnostic thresholds for clinical disorders. While psychological distress may include features of both anxiety and depression, it represents a more general reaction to stress and is not tied to specific diagnostic criteria [17, 18] Belay et al. (2021), define psychological distress as emotional suffering characterized by symptoms of depression (e.g., lack of interest, sadness, and hopelessness) and anxiety (e.g., restlessness and tension). It is often accompanied by physical symptoms, including insomnia, headaches, and fatigue, which can vary across different populations and contexts [19]. According to the Diagnostic and Statistical Manual of Mental Disorders, Fifth Edition (DSM-5), psychological distress is an undifferentiated cluster of symptoms, including anxiety and depression, functional impairments, disruptive personality traits, and behavioral challenges [20]. It reflects an individual’s psychological response to environmental stressors and adaptation and negatively affects job performance, family relationships, and overall well-being [11, 21, 22].

Nursing students have been identified as particularly vulnerable to psychological distress during the COVID-19 pandemic, even more so than students in other fields [23]. Andargeery et al. (2024) emphasize that nursing students face heightened academic and clinical stressors, compounded by concerns about their future careers. Nursing education is inherently demanding, with a rigorous theoretical curriculum, heavy workloads, strict time constraints, and intensive clinical experiences, all of which contribute to elevated stress levels. During clinical rotations, nursing students encounter patient suffering, ethical dilemmas, and the fear of making mistakes—often in high-pressure, life-and-death scenarios. These challenges significantly amplify the psychological burden they face [24]. Compared to students in non-healthcare disciplines, nursing students exhibit higher levels of psychological distress, including elevated anxiety, depression, and stress. Factors contributing to this include their direct involvement in clinical settings, fear of infection, and the emotional toll of caring for COVID-19 patients. Many also reported feeling unprepared and inadequate due to limited clinical experience in managing pandemic-related cases [25]. Even before the pandemic, studies showed that nursing students experienced higher rates of depression, anxiety, and psychological distress than the general population [26]. Research has also found that psychological issues are more prevalent among nursing students than medical students [27]. For instance, a survey conducted in Italy with over 600 nursing students revealed that more than 70% experienced high levels of psychological distress [28]. The COVID-19 pandemic led to prolonged university closures, resulting in challenges such as isolation, ineffective online learning, and difficulties in effectively conveying concepts due to limited in-person interaction [29–31]. The COVID-19 pandemic has further exacerbated these issues, profoundly disrupting their academic experiences and intensifying the mental health challenges within this group.

In nursing education, approximately 50% of training is structured to occur within hospital clinical settings [32]. However, during the pandemic, with the shift to virtual classes, clinical training was either canceled or conducted in non-COVID-19 units on a limited basis. This shift led to a decline in the quality of clinical education, raising concerns about inadequate learning, incompetence, and lack of experience among students. It also fueled anxiety about their future careers. Some students, however, remained connected to clinical environments and faced anxiety levels comparable to those of working nurses. This group included students employed in hospitals, those attending universities—such as military institutions—that continued clinical training, and students who volunteered to assist with patient care during the pandemic [33]. Several studies have indicated that the prevalence of psychological distress among nursing students during the COVID-19 pandemic ranged between 26.6% and 50% [34–36]. This heightened psychological burden can significantly impact clinical performance and potentially compromising the quality of patient care.

The psychological distress experienced by nursing students also negatively impacts the quality of care they provide to patients. It disrupts their cognitive processes and communication skills, leading to poor clinical decisions and an increased risk of medical errors [37]. Although students across various disciplines faced challenges during the pandemic, such as adapting to virtual learning and coping with social isolation, nursing students were uniquely affected due to their direct involvement in healthcare environments. This exposure significantly heightened their fear and mental strain. Overall, medical and healthcare students reported substantially higher rates of mental health issues compared to students in non-healthcare disciplines. Moreover a review highlighted that depression and anxiety were especially prevalent among healthcare students [25]. Given that nursing students frequently encounter personal, academic, and work-related stressors throughout their education, it is critical to examine how these factors interact to develop targeted interventions that address their psychological distress [38]. Nurses—constituting approximately 60% of the healthcare workforce—maintain the closest relationships with patients and play a pivotal role in health promotion and care delivery [39–41].

Our study aims to redefine psychological distress by exploring its traits, including physical and emotional symptoms, and evaluating its positive and negative consequences. The focus is specifically on nursing students, offering a nuanced understanding of this condition within the context of their unique experiences and challenges. Therefore, prioritizing the mental health of nursing students is crucial.

Given that COVID-19 has been declared no longer in a state of emergency, there remains a possibility of a new wave involving a mutated form of the virus or the emergence of new infectious pandemics. To prevent nursing students from experiencing similar levels of psychological harm in the future, it is crucial to gain a thorough understanding of the various dimensions of psychological distress they experienced during a pandemic. This knowledge will enable timely and effective interventions. Furthermore, the limited research on the antecedents and consequences of psychological distress among students—especially in critical contexts like epidemics, where the negative psychological impacts are heightened—served as a key motivation for this study. Clarifying the concept of psychological distress among nursing students is essential for achieving a clearer understanding of the term, reducing ambiguity, and minimizing the risk of misinterpretation. Conducting this study within the Iranian context with its unique social and cultural dynamics, offers the opportunity to expand and deepen insights into psychological distress. Exploring these diverse sociocultural factors may challenge existing theoretical models, thereby paving the way for their refinement or the creation of new frameworks with broader global relevance.

This study was designed to define and clarify the concept of psychological distress among nursing students during the COVID-19 pandemic. Such clarification is necessary due to the wide range of existing definitions and the importance of establishing a shared, comprehensive understanding. This understanding can be a foundation for developing targeted interventions and assessment tools. Additionally, this study contributes a unique cultural and social perspective by focusing on Iranian nursing students. Iran’s distinctive educational and societal conditions provide valuable insights that advancing theoretical models. The decision to use conceptual analysis is particularly significant, as it integrates qualitative data with evidence from literature reviews, resulting in a more comprehensive and practical definition of psychological distress.

## Method

This study combines a literature review with qualitative research to investigate psychological distress among nursing students during the COVID-19 era. The Schwartz-Barcott and Kim hybrid concept analysis model was used to clarify this concept. This model is particularly effective for examining and refining nursing phenomena, promoting the evolution and development of key concepts [42], and enhancing their application in real-world contexts by eliminating the abstract and vague nature of these concepts [43].

Schwartz-Barcott and Kim developed the hybrid model of conceptual analysis, which combines both theoretical and empirical analysis. This model is an effective method for understanding underlying concepts, serving as a reference framework that bridges academia and practice, making it especially relevant and applicable to the nursing field [44–46]. This model was chosen for its ability to clarify vague concepts and close the gap between theory and practice in nursing. The hybrid method was adopted because combining students’ perspectives with a literature review offers a more thorough and reliable understanding of the concept of psychological distress among nursing students during the COVID-19 pandemic. The model consists of three stages of theoretical, fieldwork, and a final analysis:

### Theoretical stage

In this stage, an extensive literature search was conducted to gain a thorough understanding of the concept and perform an integrated review. Throughout this process, a sense of how the concept has developed over time was established [42]. The goal of this stage was to build a solid foundation for in-depth analysis and to redefine the concept in the next stage [47].

#### Search strategy

In the theoretical stage, following the PRISMA checklist and studies utilizing various combinations of keywords such as psychological distress, depression, anxiety, nursing students, student nurses, COVID-19 pandemics, coronavirus, and their Farsi equivalents, valid and accessible international databases, including PubMed, Science Direct, Scopus, ProQuest, Google Scholar, Web of Science and National Databases SID and Magiran, were used in the period from December 2019 to June 2022. Using databases like PubMed and Science Direct in the literature search ensures a more comprehensive, credible, and efficient review process, ultimately improving the quality of the research. These platforms provide advanced search tools, such as Medical Subject Headings (MeSH) in PubMed, to support precise and targeted queries. Additionally, some offer access to full-text articles for free, expanding the availability of critical resources. The texts were thoroughly reviewed, various definitions were extracted and compared, and different aspects of the concept were examined and discussed. The process of “extraction and comparison” involved:

##### Extraction

Identifying definitions, characteristics, antecedents, and consequences of psychological distress from selected articles.

##### Comparison

Analyzing how these elements varied across studies to identify patterns and gaps.

The “various dimensions” were determined by grouping repeated themes and concepts identified through a thematic synthesis of the articles. The search strategy for this study followed the Cochrane Collaboration method.

The Cochrane Collaboration is a globally recognized network dedicated to producing, updating, and disseminating systematic reviews on therapeutic interventions (known as Cochrane Reviews). “Each Cochrane Review adheres to standardized criteria, ensuring consistency and rigor.” The process includes a predefined protocol for search strategies to identify interventional studies, along with standardized evaluation criteria for interpreting these studies.

Cochrane Reviews utilize a transparent and comprehensive literature search, incorporating databases such as Medline and Embase, along with manual searches. They adhere to a systematic and pre-established approach for assessing study quality and presenting results through meta-analysis. Key evaluation elements include study methodology, result presentation, reporting of dropouts and the identification of potential biases such as selection and reporting biases [48].

The 7-step method of the Cochrane Collaboration includes the following:

1. Defining keywords by focusing on “psychological distress” among nursing students and creating a list of terms that represent the target population and the type of model being developed.
2. Determining synonyms for keywords by identifying alternative terms and utilizing the MeSH database to define these synonyms.
3. Controlling for spelling variations in keywords and terms, or using appropriate truncation.
4. Determining the controlled vocabulary (keywords) using database indexes (MeSH for Medline, EMTREE for EMBASE).
5. Deciding whether to focus the search on individual keywords or combinations of keywords.
6. Verifying that all words are spelled correctly.
7. Systematically combining all search terms using the words “OR” and “AND” (The Cochrane Library).

The inclusion criteria consisted of all original articles written in English or Farsi that addressed the prevalence of psychological distress and its synonyms in nursing students during the COVID-19 pandemic. These articles were required to be published in peer-reviewed scientific journals. Since Farsi is the primary language of Iranian academic and cultural discourse, excluding these articles would result in the omission of valuable contributions and perspectives essential to the research. Including Farsi articles not only ensures a more comprehensive understanding but also enhances the cultural validity and relevance of the findings. The exclusion criteria included articles without access to full text, non-original articles, theses, systematic reviews and meta-analyses, letters to the editor, conference articles, and research conducted on populations other than nursing students. To ensure accuracy, two researchers independently searched for articles, conducted qualitative evaluations, and extracted the data. In total, 1945 articles were found, and after removing duplicates and applying the inclusion and exclusion criteria, 60 articles were selected for data extraction. Two researchers, each independently reviewing the inclusion and exclusion criteria, reviewed the title, abstract, and main text of the articles. After completing these steps, the data were extracted and summarized, with the article title, author names, year of publication, sample size, and the population, research location, tools, and results organized in a tabular format serving as a checklist.

#### Data synthesis of the theoretical part

All articles were reviewed line by line. In many cases, the texts were read independently by two authors. In case of a disagreement, a third author was consulted for clarification. Subsequently, a definition matrix for the psychological distress of nursing students was created, categorized into three groups: antecedents, characteristics, and consequences. Finally, concepts related to each category were extracted and organized accordingly.

### Work in the field

In this stage, the concept developed in the first stage was strengthened,, integrated, and refined through fieldwork. This stage may overlap with the first stage in its timeline [42].

#### Setting up the field and entering the interview

To conduct a qualitative study, targeted sampling was applied to nursing students from the Army Nursing Faculty. The inclusion criteria for interview participants were as follows.

The study included nursing students in the 3rd to 8th semesters during the COVID-19 pandemic, from both genders, varying ages, and diverse living situations (dormitory or private housing). The exclusion criteria were students who were unwilling to participate or unable to complete the interview process.

To maintain maximum diversity among participants, students from different academic semesters, of both males and females, living in private homes or dormitories, and of various ages were considered. The interviews were conducted by the researcher, who was a psychiatric nurse, to ensure participants felt comfortable discussing sensitive topics. The interviews continued until data saturation was reached, and no new information emerged. Before starting the study, the purpose was explained to the participants, and informed consent was obtained. The location of the interviews was chosen based on the participant’s preference. The interviews were recorded using an MP3 player with the participant’s consent. A semi-structured interview format was used, guided by general questions such as *Please describe your day at university and what changes you think COVID-19 has caused for you. What strategies did you use to calm yourself down during the pandemic? What challenges did COVID-19 create for you and your educational situation?* Probing questions were used to clarify most of the responses. The interviews lasted from 15 to 30 min. At the end, the researcher provided participants with contact information in case they wished to follow up or complete the interviews. The interview guide and protocol are reported in Appendix 1.

#### Data analysis of the qualitative section

Immediately after the interview, the researcher carefully listened to the recordings several times, transcribed them by hand onto paper, and manually coded the data. Additionally, the Elo and Kyngäs’ methods were employed for directed content analysis.

This approach involves a structured process that allows researchers to analyze qualitative data within a predefined framework, while remaining open to the emergence of new themes. The steps followed in this analysis are outlined below:

##### Data familiarization

The first step involved repeatedly reading the interview transcripts to gain a thorough understanding of the content and ensure a deep comprehension of the data.

##### Initial coding

Guided by the theoretical framework informed by the literature review, initial codes were developed to capture key aspects of the data. These codes were grouped into three main areas: antecedents, characteristics, and consequences of psychological distress.

##### Code refinement

As the analysis progressed, the codes were continuously refined and expanded. New codes emerged, while some initial codes were combined or further specified. This iterative refinement ensured the final coding framework accurately reflected the data and addressed the research questions.

##### Category formation

After refining the codes, the researchers organized them into subcategories, which were then classified under the three main categories (antecedents, characteristics, and consequences). This step provided a more structured approach to interpreting the data.

##### Interpretation

In the final stage, the coded data were analyzed in the context of the theoretical framework. This involved comparing the categories and subcategories that emerged from the interviews with concepts identified in the literature review. The aim was to integrate empirical data with theoretical insights to deliver a comprehensive understanding of psychological distress among nursing students.

Elo and Kyngäs (2008) highlight that directed content analysis offers a systematic and transparent approach for analyzing qualitative data, while maintaining the flexibility to incorporate emerging insights. This approach was pivotal in guiding the interpretation of our interview data and in connecting it to the existing body of knowledge on psychological distress [49].

#### Accuracy and scientific validity of the study in the qualitative phase

An important aspect of qualitative studies is their credibility, scientific rigor, and precision. According to Guba and Lincoln, the four criteria for rigorin qualitative research are credibility, transferability, dependability and conformability [50]. In this study, to meet these criteria during the qualitative phase, sufficient time was allocated for data collection and analysis, as well as integration (data integration, researcher integration, researcher triangulation, and methodological triangulation). Various methods were used to gather data (semi-structured interviews and field notes), and feedback from supervisors and advisors was incorporated. Additionally, the manuscripts were shared with participants for member checking, and the data analysis and coding process were reviewed by the research team to enhance dependability.

#### The final analysis stage

This stage combines theoretical analysis with insights from practical observations and the reporting of findings [47]. In this phase, the researcher compared the definitions and characteristics of the concept, which were derived from both the theoretical phase and fieldwork, integrating and synthesizing these results. To align the findings from the literature and interviews, the following steps were undertaken:

Codes extracted from the interviews were aligned with the antecedents, characteristics, and consequences identified in the literature. A comparison was conducted to identify overlapping themes as well as new or contradictory insights. Unique codes from the interviews were either incorporated into existing categories or used to develop new subcategories. This integrative approach facilitated a comprehensive understanding of psychological distress.

## Results

### Theoretical phase

The characteristics of the reviewed articles are detailed in Supplementary Material Table 1. The author, year of the publication, country, sample size, and survey results—categorized into characteristics, antecedents, and consequences were extracted from the articles and presented in Supplementary Material Table 1.

The reviewed studies provided diverse perspectives on psychological distress. For instance, certain studies emphasized personality traits (such as introversion and perfectionism) as precursors, while others concentrated on external stressors including academic workload. These varying viewpoints enriched our understanding of psychological distress its highlighting intricate and multifaceted nature.

As a result of analyzing the articles included in the study, the definition of psychological distress among nursing students was derived from three categories: antecedents, characteristics and consequences.

#### Antecedents of psychological distress

Antecedents refer to the factors that precede and contribute to the psychological distress experienced by nursing students. These factors are categorized into several subcategories:

##### Personality and demographic factors

This subcategory includes variables such as income, place of residence, gender, education year, age, marital status, and various personal factors, including work status, medical history, and chronic diseases. Additionally, traits such as self-awareness, exposure to violence, and smoking habits are identified as key contributors. For instance, students experiencing higher stress due to their work situation or poor eating habits reported greater psychological distress.

##### Social factors

This subcategory includes the influence of family environment, organizational support, and family support. Additionally, social stigma and feeling of loneliness were identified as significant stressors. For instance, students reported increased psychological distress when they were isolated due to a lack of social connections or faced negative social stigma related to the COVID-19 situation.

##### Psychological factors

Factors such as self-esteem, emotional exhaustion, and uncertainty were identified as key contributors to psychological distress. Many students reported experiencing emotionally exhaustion and uncertainty about their future careers, which significantly heightened their anxiety and distress levels.

##### Physical factors

Students with health problems, such as being overweight, or those lacking a self-care plan or a healthy lifestyle, reported experiencing higher levels of psychological distress. These physical concerns exacerbated stress levels and adversely affected students’ mental health.

##### Educational factors

The transition to online learning and the challenges of adapting to this new educational environment significantly contributed to psychological distress. Senior nursing students, as well as those concerned about their academic progress, found this shift particularly demanding.

##### COVID-19 pandemic context

Exposure to the virus, whether through family members, close contact with infected patients, or personal health concerns, significantly contributed to psychological stress. Factors such as quarantine, fear of infection, and the loss of loved ones were particularly distressing.

#### Characteristics of psychological distress

Psychological distress manifests in various ways, which can be categorized into physical, emotional-psychological, and intellectual symptoms:

##### Physical symptoms

Common physical manifestations include headaches, back pain, stomach aches, sleep disturbances, weight gain, and fatigue. Many students reported experiencing physical exhaustion and a general decline in their overal health as a result of constant emotional and mental strain.

##### Emotional-psychological manifestations

The distress resulted in intense negative emotions such as anxiety, stress, fear, and depression. Many students experienced feelings of worthlessness, loneliness, irritability, and even symptoms of PTSD. The mental toll of the pandemic eroded motivation, triggered emotional breakdowns and significantly impacted their overall mental well-being.

##### Intellectual manifestations

Cognitive symptoms were also prevalent, including uncontrollable worry, difficulty concentrating, and a high frequency of negative thoughts. Students reported a persistent sense of impatience and trouble focusing on their academic work, which further intensified their stress and hindered their performance.

#### Consequences of psychological distress

The consequences of psychological distress were both negative and positive, underscoring the complex impact of the pandemic on the lives of nursing students.

##### Positive coping mechanisms

Some students managed their distress by practicing self-care, engaging in worship, and strengthening their connection with spirituality. Many also reported improvements in their psychological well-being and family relationships. These positive coping strategies enabled them adapt to the challenges of the pandemic and maintain a sense of mental balance.

##### Drop in academic performance

A common outcome was a decline in academic performance. Many students found it challenging to adjust to online learning, encountering issues such as difficulty focusing and academic setbacks, which future heightened their distress.

##### Decreased quality of life

Students reported a decrease in life satisfaction and overall health during the pandemic. They experienced reduced well-being, and many continued to face long-term effects on their daily lives.

##### Social harm

Social isolation, exacerbated by pandemic restrictions, caused significant harm to students’ social. Many turned to internet addiction, which further reduced their social engagement. Feeling of loneliness were common, and maintaining meaningful relationships during the crisis become increasingly difficult.

### Fieldwork phase

The participants in this study were 24 nursing students with an average age of 21.87 years. Detailed demographic information is provided in Supplementary Material Table 3. During the analysis phase, 532 codes were extracted from the 24 interviews. The authors also defined psychological distress among nursing students, categorizing it into three main categories: antecedents, characteristics, and consequences (Table 1).

**Table 1:** Psychological distress of nursing students in three main categories: antecedents, characteristics, and consequences in the interview phase

An example of codes	Subclass	Subcomponent		Main component
Income, place of residence, gender, year of education, age, marital status, level of education, economic status of the family	Demographic factors	Antecedents		**Psychological distress of nursing students in the COVID-19 crisis**
Nonobservance with health protocols, spreading rumors, lack of entertainment, suspension of gatherings and religious and cultural centers, quarantine, hearing negative news, social stigma	Social factors			
Fear of infection and death of oneself and family, self-esteem, emotional exhaustion, low mood, desire to be alone-realism-humor-positive attitude-acceptance-compatibility-attention to meaning	Psychological factors			
University support-family support-social support	Support systems			
Overweight, disease history, genetic history, self-care plan, being a smoker, type of sleeping and eating pattern	Physical Health Concerns			
Challenges of virtual education - lack of preparation of professors, - insufficient educational infrastructure, inappropriate educational environment, possibility of cheating, low attention of professors to class attendance, Ignorance of students about the way of evaluation, - allocating little time to answer questions - lack of regular and timely attendance of professors and students during classes, inappropriate educational platform, neglecting practical classes, Not feeling like a student, the phone’s battery is running out, the phone’s antenna is weak, not familiar with the required programs and software	Educational Challenges			
Positive covid in family members, encountering a covid-infected patient, lack of personal protective equipment, fear of spreading the infection, quarantine, death of family members, relatives or friends due to covid-19	COVID-19 Pandemic Context			
Pain in the head, neck, stomach and back, sleep disorders	Physical manifestations	Characteristics		
Negative emotions, depression, anxiety, stress, fear, pressure, sadness, anger, depression, monotony, loneliness, irritability, obsession	Emotional, psychological manifestations			
Uncontrollable worry, concentration problems, negative thoughts, impatience	Intellectual demonstration			
Relaxing activities - enjoyable activities, exercise, avoiding following negative news, distraction, talking with friends about problems, gratitude, self-care, worship and spirituality.	Positive coping Mechanisms	Post-Traumatic Growth (PTG)	Consequences	
Maturity, resilience, family friendship, sacrifice	Character development			
Increasing study, updating information - access to classes at all times, - the possibility of working students participating in classes, - access to rare books and pamphlets - acquiring technical skills. (computer)	Improving academic performance			
Reducing the cost of going to the university - reducing the cost of buying clothes - reducing the consumption and purchase of stationery - extra work	Economic Growth			
Difficulty in adapting to new teaching and learning methods, academic failure. Weakening of learning, lack of concentration, drop in grades, reduction of teacher‒student interaction	Drop in academic performance			
		Adverse Outcomes		
Tension with family, tension with friends, social isolation, addiction to internet, decrease in social vitality	Social harm			
Decreased general health level, physical pain, vision disorder, weight gain, physical fatigue, decreased physical activity, insomnia, sleep and eating disorder.	Physical injuries			
Decrease in psychological well-being, decrease in life satisfaction, weakening of will, decrease in motivation	Psychological injuries			
Decreased job satisfaction, negative perception of the work environment, job instability	Occupational complications			

#### Antecedents of psychological distress in nursing students

##### Demographic factors

The demographic characteristics of nursing students significantly influenced theire experiences of psychological distress during the COVID-19 pandemic. Female students, those residing in dormitories, younger individuals, and unmarried students reported more severe symptoms of psychological distress. This pattern can be attributed to several interrelated factors: the increased isolation experienced by students living away from their families, the heightened emotional vulnerability often associated with younger individuals, and the added challenges unmarried students face in managing academic demands without the emotional and logistical support of a family.

Additionally, extended years of education seemed to amplify the burden, as students struggled with increasing academic and personal expectations. This complex relationship between demographic variables and psychological distress highlights the dual role of social and familial structures—some acting as protective factors, while others, such as gender and living arrangements, exacerbating feelings of anxiety and isolation.

For instance, Participant 4 shared a poignant account of how his family’s economic difficulties compounded his psychological distress. He described the stress he endured after his father, a construction worker, lost his job during the pandemic, stating, *“My father was a construction worker; he was not offered any job in Corona.”* This testimony underscores the significant impact of family financial struggles on students’ mental health, illustrating how economic strain can become a persistent source of stress, fueling feelings of helplessness, frustration, and uncertainty about the future.

##### Social factors

Social factors, such as the stigma surrounding COVID-19, emerged as significant contributors to psychological distress. In particular, the social stigma tied to vaccination and disease transmission added another layer of stress for nursing students. Participant 3 shared their experience: *“The university told us to get vaccinated*,* but when I mentioned it at home*,* I faced a negative reaction from my family*,* saying*,* ‘Now you’ll get COVID and spread it to others.’”* The fear of judgment from close social circles, especially family members, heightened students’ emotional distress. This stigma further isolated them, making it more difficult to seek support and exacerbating the social and emotional challenges of the pandemic.

##### Support systems

Support systems played a crucial role in either alleviating or exacerbating psychological distress. Students with robust family, social, and institutional support reported fewer symptoms of distress, underscoring the vital role of support networks in mitigating the effects of external stressors. One participant remarked, *“Even providing masks*,* gloves*,* and disinfectants from the faculty would make us feel better.”* This straightforward yet impactful gesture from the university not only offered tangible health protection but also conveyed a sense of care and unity, highlighting how institutional support can greatly bolster psychological resilience during crises.

##### Psychological factors

Psychological factors were another critical antecedent of distress. Negative thought patterns, such as persistent feelings of hopelessness and pessimism, were frequently mentioned by participants. One participant described the emotional toll of the pandemic, saying, *“Someone always said in my mind: we all die eventually; why are you fumbling around?”* This remark underscores how deeply ingrained existential fears and negative thinking can intensify emotional and psychological distress. Moreover, participants with pre-existing mental health conditions or a history of psychological challenges were particularly vulnerable, as the pandemic worsened their symptoms. These findings underscore the critical need for tailored mental health interventions for students with a history of psychological disorders.

##### Physical health concerns

Physical health concerns also significantly influenced the psychological well-being of nursing students. Participants with underlying health issues or heightened vulnerability to the virus experienced elevated anxiety about their health and the potential consequences of contracting COVID-19. Participant 2 remarked, *“We as a whole family get sick easily. I am prone to getting the virus*,*”* illustrating how worries about susceptibility to illness served as a persistent source of distress. This anxiety was especially acute among students with chronic health conditions, leaving them feeling more at risk both physically and emotionally.

##### Educational challenges

Educational challenges emerged as a significant contributor to psychological distress among nursing students. The abrupt transition to virtual learning brought a range of difficulties, affecting both students and faculty. Inadequate preparation, limited technological infrastructure, and a lack of support systems made adapting to online education particularly stressful. This shift left students feeling confused, frustrated, and helpless, leading to disengagement and reduced motivation. One participant shared, *“Virtual education was very dumb for both us and our professors. Because we were not present in class*,* we had no motivation to learn.”* The absence of in-person interactions caused students to feel disconnected from peers, professors, and academic material, further heightening stress and anxiety. Many students also expressed concerns that these challenges would compromise their education and future professional competence.

##### COVID-19 pandemic context

The unique context of the COVID-19 pandemic introduced additional stressors that heightenend psychological distress among nursing students. A pervasive fear of infection, both for oneself and loved ones, was a constant source of anxiety, especially during the early stages of the pandemic. Quarantine measures further intensified these feelings, particularly for students unable to visit their families. Participant 13 recounted, *“I did not go home for several months due to being quarantined at the university during the coronavirus period; I was very homesick.”* The prolonged separation from loved ones, combined with the uncertainty surrounding the pandemic, deepened emotional distress, contributing to feelings of isolation, sadness, and homesickness.

#### Characteristics of psychological distress in nursing students

During the COVID-19 pandemic, nursing students experienced significant psychological distress that manifested as a range of emotional, cognitive, and physical symptoms, highlighting the multifaceted challenges they encountered.

##### Emotional-psychological symptoms

Emotional and psychological symptoms were among the most frequently reported manifestations of distress, encompassing anxiety, stress, fear, anger, despair, obsession, and depression. These emotions were deeply intertwined with the overwhelming and unpredictable circumstances of the pandemic.

###### Anxiety and stress

Anxiety and stress were pervasive among students, often stemming from the uncertainty surrounding the pandemic, fears about their own health and the health of loved ones, and the rapid, disorienting changes in their academic and social environments. One participant (P11) described their experience, stating: *“Life had no meaning for me; it was all repetition*,* and I had become monotonous.”* This expression captures the emotional numbness and existential dread many students endured as the pandemic disrupted their routines and intensified feelings of uncertainty.

###### Fear

Fear emerged as another prominent emotional response, primarily driven by concerns about contracting the virus or inadvertently transmitting it to family members. This heightened sense of fear magnified emotional distress, leaving students feeling vulnerable and powerless. Some students expressed fears of not receiving timely medical care or witnessing loved ones suffer from severe illness, further amplifying their distress.

###### Anger and despair

Anger and despair were significant emotional responses reported by students. Anger often stemmed from frustration with the restrictions imposed during the pandemic, including the abrupt transition to online learning, diminished social interactions, and a lack of clear direction. Conversely, despair arose from a sense of lost control over their lives and future. This combination of emotions was exacerbated by the prolonged state of crisis, contributing to a deep sense of helplessness and a decline in overall well-being.

###### Obsessions and depression

Obsessive thoughts represented another facet of psychological distress. Many students reported being trapped in repetitive, negative thought cycles. One participant (P3) explained, *“After COVID-19*,* I was obsessed with thinking. If someone told me something*,* I would fight with myself for days.”* This tendency to ruminate over perceived slights or hypothetical scenarios underscored the overwhelming nature of their cognitive struggles. Depression was also prevalent, with students expressing feelings of sadness, hopelessness, and a lack of motivation to engage in previously enjoyable activities. These symptoms highlighted the profound emotional toll the pandemic took on their mental health.

##### Intellectual symptoms

Intellectual symptoms, including confusion, cognitive overload, and intensified intellectual conflict, were particularly pronounced during the pandemic. Nursing students, accustomed to rigorous and hands-on education, encountered substantial challenges with the sudden shift to virtual learning, which introduced a range of intellectual difficulties.

###### Cognitive overload and confusion

Many students reported feeling overwhelmed by the sheer volume of information they were required to process and retain in the new virtual learning environment. This cognitive overload often resulted in confusion, difficulty concentrating, and a pervasive sense of mental fatigue. Although online education offered convenient access, it also fostered a sense of disconnect between students and instructors, making the learning experience feel impersonal and less effective.

###### Intellectual conflict

Another frequently reported intellectual symptom was internal conflict, as students grappled with doubts about their academic abilities, the relevance of their education, and their future professional prospects. The absence of face-to-face interaction and the sudden shift to remote learning generated uncertainty regarding the quality of their education. One participant shared their struggle: *“After COVID-19*,* I was obsessed with thinking. If someone told me something*,* I would fight with myself for days”* (P3). This statement reflects the deep mental conflict students experienced as they struggled to reconcile their existing knowledge and expectations with the new realities brought on by the pandemic.

This intellectual conflict frequently gave rise to self-doubt, with students questioning their academic competence and their ability to succeed in their chosen profession. This growing uncertainty contributed to feelings of despair and helplessness. The combination of emotional distress and internal intellectual struggles created a feedback loop, amplifying the adverse effects on students’ mental health mental health.

##### Physical symptoms

Psychological distress also presented as physical symptoms, with sleep disturbances identified as one of the most commonly reported issues. The interaction between emotional strain and intellectual challenges significantly disrupted students’ ability to maintain regular sleep patterns.

###### Sleep disorders

Sleep disturbances were a frequent consequence of the pandemic’s emotional and intellectual toll. Anxiety, stress, and obsessive thoughts often frequently disrupted students’ ability to sleep, leading to insomnia or irregular sleep schedules. One participant (P8) described their experience: *“My sleep was disturbed; I was awake at night and asleep in the morning.”* This irregular sleep pattern was a widespread issue among students and underscored the profound impact of psychological distress on their physical well-being.

The disruption in sleep patterns not only mirrored students’ distress but also amplified it, contributing to fatigue, reduced concentration, and a further decline in overall health. Sleep deprivation had a cyclical effect on their psychological well-being, as poor sleep worsened mood and heightened anxiety and stress levels. Additionally, inadequate rest impaired cognitive function and memory, compounding the intellectual difficulties associated with virtual learning. The cumulative impact of sleep deprivation on emotional regulation, cognitive performance, and physical health underscored its role in perpetuating a vicious cycle of distress.

#### Consequences of psychological distress experienced by nursing students

The consequences of psychological distress among nursing students during the COVID-19 pandemic were both adverse and beneficial, emphasizing the complex and multifaceted nature of this phenomenon. These outcomes offer valuable insights into how distress influenced various dimensions of students’ lives while also shedding light on their resilience and coping strategies.

##### Adverse outcomes

###### Decreased academic performance

A significant negative consequence of psychological distress was a decline in academic performance. Students reported that the overwhelming nature of their distress adversely affected their motivation and ability to focus on their studies. For example, one participant remarked, *“I was so depressed that I did not feel like going to my handouts at all. Studying had become meaningless to me”* (P1). This lack of academic drive not only resulted in lower grades but also diminished overall learning outcomes. The combination of low motivation and feelings of hopelessness posed substantial barriers for students in maintaining academic progress.

###### Social isolation and increased conflict

Social distancing measures necessitated by the pandemic heightened feelings of social isolation among nursing students. Participants reported a sense of disconnection from their peers and families, which further exacerbated their psychological distress. One participant stated, *“I was addicted to my phone; even when I did not have class*,* I used to surf the web all the time”* (P10). Excessive screen time, while a coping mechanism, often intensified feelings of loneliness and detachment. Additionally, isolation contributed to heightened tension within families and strained interpersonal relationships, highlighting the broader social implications of psychological distress.

###### Psychological damages

Psychological distress had profound consequences on students’ mental health. Many described feelings of emptiness and despair, along with a pervasive dissatisfaction with life. One participant stated, “*I was always thinking about death*,* and I did not feel any joy in life”* (P7). These emotional burdens were deeply rooted in fears of infection, the potential loss of loved ones, and uncertainty about the future, significantly diminished students’ quality of life and causing long-term psychological harm.

###### Physical health consequences

Distress also manifested in physical symptoms. Changes in eating habits, such as overeating, were common coping strategies. *“Whenever I get anxious*,* I start to overeat. During this time*,* I overeat so much that I gained a few kilos”* (P11). Weight fluctuations and other physical ailments negatively impacted overall health, illustrating how psychological distress extends beyond mental health to affect physical well-being.

###### Negative impact on professional perception

Psychological distress also Influenced students’ perceptions of their future profession. One participant admitted, *“I got sick of nursing; I think it is a troublesome and stressful job”* (P15). The challenges and emotional toll of the pandemic led some students to question their commitment to nursing, potentially impacting their long-term engagement with the profession.

##### Post-traumatic growth (PTG)

###### Coping mechanisms

Despite the negative consequences, some students were able to transform their distress into opportunities for growth. By engaging in self-care strategies, they built resilience and gained a deeper understanding of themselves and their relationships. Humor emerged as a significant coping mechanism; for example, Participant 13 shared, *“Whenever my pressure gets too high*,* I laugh*,* joke*,* and make fun of [myself].”* Additionally, social connections and spiritual practices played vital roles in fostering growth. Participant 1 noted, *“After corona*,* I appreciated my friends more and enjoyed hanging out with them*,*” while Participant 17 explained*,* “Whenever I felt low*,* I started to pray.”* These strategies not only alleviated distress but also facilitated personal growth and strength.

###### Character development

Interestingly, some students reported improved academic performance despite experiencing psychological distress. The transition to virtual education, although initially challenging, enabled students to better manage their academic responsibilities. Online classes eliminated logistical barriers such as commuting and provided easier access to educational resources. As Participant 21 remarked, *“One of the benefits of virtual education was having access to professors’ materials*,* including pamphlets and even books that were otherwise hard to find.”* These changes allowed some students to thrive academically during the pandemic.

###### Improved academic performance

Interestingly, some students experienced improvements in academic performance despite their distress. The shift to virtual education, thought initially challenging, allowed students to better manage their academic responsibilities by removing. logistical barriers like commuting and enabled easier access to resources. Participant 21 noted, *“One of the positive points of virtual education was access to professors’ pamphlets*,* even books that were not easy to find.”* These changes helped some students excel academically during the pandemic.

###### Economic growth

For some students, the pandemic unexpectedly brought about economic opportunities. The demand for healthcare workers allowed nursing students to work in healthcare settings, providing financial relief and professional growth. Participant 7 shared, *“At that time*,* due to the special circumstances of Corona*,* I*,* a final semester nursing student*,* was allowed to work in private hospitals with good financial benefits.”* Additionally, the closure of educational institutions reduced commuting costs, as Participant 12 observed: *“Every time I came from our city to the university*,* I had to pay a lot of money. Closing the university and not commuting was economically beneficial for me.”* These financial benefits offered a sense of stability during uncertain times.

### The final analysis phase

In the final analysis phase, the findings from the theoretical phase and the fieldwork phase, including interviews, were juxtaposed and compared. Table 2 summarize the outcomes of this comparative analysis. The comparison between the theoretical and interview stages underscores the evolving understanding of psychological distress among nursing students. The theoretical phase offered a broad, framework-based perspective, whereas the interview phase provided a more nuanced and personalized insight. Table 3 presents the definition of psychological distress in nursing students during the COVID-19 crisis, based on the theoretical stage and Table 1 presents the Psychological distress of nursing students in three main categories: antecedents, characteristics, and consequences in the interview phase.

**Table 2:** Integration of the definition of psychological distress of nursing students obtained from the theoretical stage and interviews

Antecedents		Characteristics		Consequences	
Theoretical stage	Interview stage	Theoretical stage	Interview stage	Theoretical stage	Interview stage
Personality, demographic factors	Demographic factors	Physical manifestations	Physical manifestations	Positive coping mechanisms	Positive coping Mechanisms
Social factors	Social factors	Emotional, psychological manifestations	Emotional, psychological manifestations	Drop in academic performance	Character development*
Psychological factors	Psychological factors	Intellectual demonstration	Intellectual demonstration	Decreased quality of life٭	Improving academic performance٭
Physical factors	Physical Health Concerns			Social harm	Economic Growth*
Educational factors	Educational Challenges				Drop in academic performance
COVID-19 Pandemic Context	COVID-19 Pandemic Context				Social harm
	Support systems٭				Physical injuries*
					Psychological injuries*
					Occupational complications٭

*Aspects distinct between the two phases

**Table 3:** Definition of the concept of psychological distress in nursing students in the COVID-19 crisis resulting from the theoretical stage

An example of codes	Subclass	Subcomponent	Main component
Income, place of residence, gender, year of education, age, marital status, education level, work unit, work experience, chronic disease, medical history, student work status, acceptance, attention to meaning, self-knowledge, violence, strong relationship with God, being a smoker, sleeping and eating pattern, working long hours, not wanting to exercise, consuming unhealthy food	Personality, demographic factors	Antecedents	**Psychological distress of nursing students in the COVID-19 crisis**
Family climate, organizational support, family support, hearing negative news. Social stigma, feeling loneliness	Social factors		
Self-esteem, emotional exhaustion, feeling of uncertainty, feeling of coherence	Psychological factors		
Overweight, medical history, self-care plan, healthy lifestyle	Physical factors		
Structured learning environment, being a senior nursing student, worrying about academic progress, adapting to the new educational method, feeling satisfied with student life, being interested in the nursing profession.	Educational factors		
Positive covid in family members, exposure to an infected patient, lack of personal protective equipment, fear of infection, quarantine, death of family members, relatives or friends due to covid, belonging to at-risk groups.	COVID-19 Pandemic Context		
Headache, stomach and back pain, pain, sleep disorders, eating disorders, early fatigue, weight gain, decreased physical activity	Physical symptoms	Characteristics	
Negative emotions, feelings of depression, anxiety, stress, fear, feelings of pressure, feelings of worthlessness, PTSD, feelings of sadness, irritability, anger, decreased motivation, weakening of the will, feelings of loneliness	Emotional, psychological manifestations		
Uncontrollable worry, concentration problems, negative thoughts, impatience	Intellectual demonstration		
Self-care, worship and spirituality, psychological well-being, promotion of family relationships	Positive coping mechanisms	Consequences	
Difficulty in adapting to new ways of teaching and learning, weakening of learning, lack of concentration, academic failure	Drop in academic performance		
Decrease in life satisfaction, decrease in well-being, decrease in general health level	Decreased quality of life		
Social isolation, addiction to internet, reduction of social vitality	Social harm		

#### Comprehensive definition of psychological distress in nursing students during the COVID-19 pandemic

**Fig. 1:**
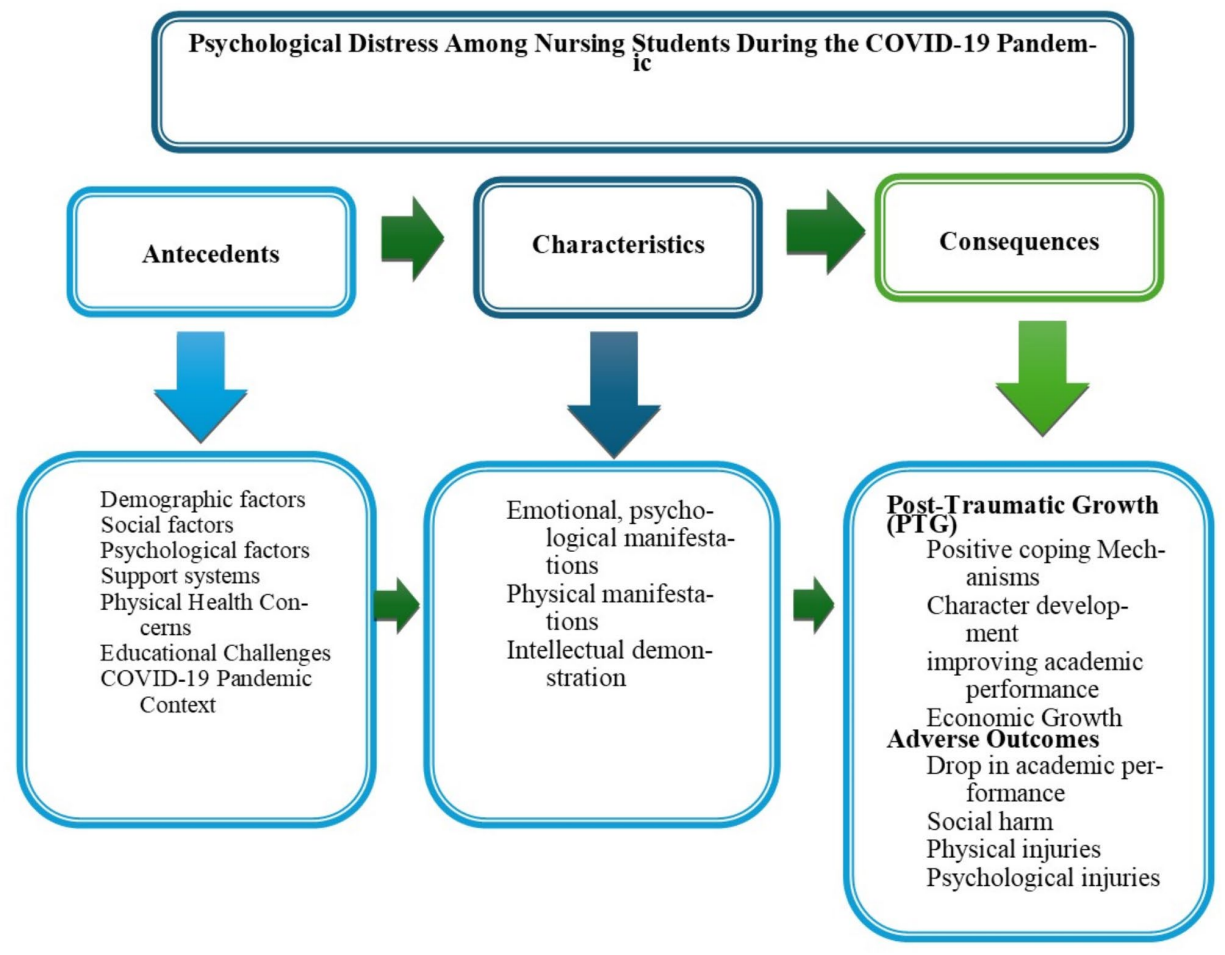
Psychological distress among nursing students during the COVID-19 Pandemic

Psychological distress in nursing students during the COVID-19 pandemic can be described as a complex, multifaceted condition influenced by the interplay of personal, social, educational, and contextual factors. It is characterized by a combination of emotional, psychological, physical, and cognitive difficulties, including anxiety, stress, sleep disturbances, and academic struggles. While this distress often led to adverse outcomes such as academic setbacks, social isolation, and emotional exhaustion, many students also experienced personal growth, resilience, and enhanced coping skills. This dual nature highlights that although the pandemic presented substantial challenges, it also provided opportunities for development and transformation, underscoring the critical role of support systems and effective coping strategies in addressing psychological distress (Fig. 1).

## Discussion

This study aimed to explore and understand the psychological distress experienced by nursing students during the COVID-19 pandemic using a hybrid study design. By integrating qualitative research conducted within the cultural context of Iran with global findings from other studies, the research offers a comprehensive perspective on the issue. The results shed light on the diverse antecedents, characteristics, and consequences of psychological distress, providing valuable insights into the intricate interplay of personal, educational, and contextual factors.

Our definition aligns with existing frameworks that conceptualize psychological distress as as a multidimensional construct spanning emotional, cognitive, physical, and social domains. Consistent with prior models (e.g., Kessler et al., 2002 [17]; Belay et al., 2021 [19]), we identify psychological distress as a response to stressors, marked by symptoms such as anxiety, depression, and functional impairments. However, our study situates this distress within the specific context of nursing education during a global health crisis, highlighting how this unique environment amplifies vulnerability.

The study identified various factors influencing psychological distress, including personality traits, social stigma, and educational challenges. Personality traits such as emotional exhaustion and negative thinking emerged as significant precursors to psychological distress. In this context, Flesia et al. (2023) reported that being female, older, less educated, living in urban areas, and being married were associated with higher levels of psychological distress during the COVID-19 pandemic [10]. In contrast, our finding suggest that female gender, younger age, and being single are more strongly linked to elevated psychological distress. One notable factor is the nature of COVID-19 itself and the unique societal conditions it created. Fear, anxiety, concern about infection, and social stigma significantly impacted nursing students, often causing them to withdraw from social interactions. Bigelsen and Schupak further argue that social isolation can exacerbate maladaptive fantasies, encouraging withdrawal into an inner world and worsening psychological well-being [51]. Consequently, social isolation exacerbating maladaptive fantasies is that prolonged periods of isolation can amplify negative cognitive patterns. also social isolation may give rise to adverse cognitive processes, which in turn can increase the likelihood of maladaptive behaviors.

Perceived stress was identified as another significant contributing factor. It refers to an individual’s assessment and interpretation of their ability to handle stress effectively. When individuals perceive stressors as overwhelming and catastrophic and struggle to regulate their emotions, it sets the stage for psychological distress [52]. During the COVID-19 crisis, the enforced isolation, which deeply impacted individuals’ social, economic, and professional lives, led to heightened feelings of loneliness, anxiety, and stress, becoming a significant precursor to psychological distress. In this context, Jahangiri et al. demonstrated this impact on nurses during the COVID-19 period [53]. This study is consistent with the findings of our research, suggesting that psychological distress ultimately leads to negative outcomes, including disruptions in daily and occupational functioning. Similarly, Alizadeh et al. (2020) highlighted that the nature of the disease itself was a key factor contributing to psychological distress among healthcare workers during the pandemic [11, 54]. Our study further corroborates these findings, revealing that the psychological distress caused by the COVID-19 pandemic led to negative social, physical, and academic consequences among nursing students.

The unique educational circumstances faced by nursing students, which transitioned from traditional teaching methods to online learning during the COVID-19 pandemic, emerged as a significant factor contributing to their psychological distress [55]. As nursing students were required to complete both practical and theoretical courses, quarantine measures hindered their ability to engage in hands-on training. Challenges during this period included inadequate infrastructure, high internet costs, a lack of e-learning policies and implementation strategies, insufficient technical training for staff, and the time-intensive process of preparing educational content. In a study by Heydari et al., e-learning, compounded by the fear of losing an academic year during the pandemic, was found to induce psychological distress in nursing students [56]. This study also identified physical factors as precursors to psychological distress, including disruptions to sleep and eating patterns. In this context, Brouwer’s research demonstrated that sleep disorders among nursing students during the pandemic increased their vulnerability to psychological distress [15]. Our study similarly identified physical factors as key antecedents or influential contributors to psychological distress among nursing students during the COVID-19 pandemic.

In this study, psychological distress was characterized by physical, psychological, and mental manifestations. Similarly, qualitative research by Galedar et al. found that nurses in Iran during the COVID-19 pandemic experienced psychological distress, presenting as anxiety, fear, emotional turmoil, and obsessive thoughts [57]. In a phenomenological study, Negash et al. identified symptoms of psychological distress among students, including anxiety, fatigue, headaches, and feelings of hopelessness [58].

Psychological distress among nursing students has led to various adverse outcomes, including reduced academic performance, social harm, physical and psychological damage, and occupational challenges.One notable social consequence of psychological distress was internet addiction, which emerged as a coping mechanism. Escaping mentally from a troubling external reality or seeking distraction by immersing oneself in the virtual world was observed as an avoidance response to COVID-19, manifesting as internet addiction in individuals experiencing psychological distress [59].

While psychological distress resulted in several negative outcomes, such as diminished academic performance, social isolation, and physical health challenges, it also led to positive experiences for some students. Notably, some students exhibited Post-Traumatic Growth (PTG), which encompasses personal strength, a shift in priorities, and a deeper appreciation for life and relationships. As defined by Tedeschi and Calhoun [60], PTG refers to personal growth and positive psychological changes that emerge from overcoming significant adversity or trauma. Identifying PTG could be valuable in designing future mental health interventions, resilience training, and coping strategies tailored for nursing students.

In this study, nursing students who experienced psychological distress during the COVID-19 pandemic demonstrated characteristics aligned with PTG, such as heightened resilience, greater self-awareness, and an increased appreciation for life and interpersonal connections. This growth was not simply a byproduct of overcoming distress; rather, it represented a profound transformation born from the challenges of the pandemic. The ability to derive strength and meaning from adversity underscores the potential for positive outcomes, even amidst psychological turmoil.

Students facing psychological distress employed various self-care strategies to alleviate their struggles and empower themselves to navigate the critical conditions of the COVID-19 pandemic. One culturally significant coping mechanism in Iran was prayer and worship. In this context, Rahim Karimi’s research revealed that teaching mental peace grounded in prayer and patience positively impacted feelings of failure and enhanced tolerance for distress among nursing students [61].Our study identified worship, spirituality, and a deep connection with God as both antecedents and outcomes of psychological distress. Moreover, effective coping behaviors during periods of psychological distress have been shown to significantly enhance psychological well-being [62].

Furthermore, Brouwer and colleagues found that self-care behaviors among nursing students during the pandemic had a significant negative correlation with psychological distress. They recommended incorporating these behaviors into the nursing curriculum [15]. Given the existing gap in the teaching of self-care behaviors in the nursing curriculum in Iran, it is crucial for educational planners to address this deficiency and implement strategies to cultivate these essential skills [32].

The study identified notable differences between the theoretical phase and fieldwork findings. While the theoretical phase provided a broad framework, the fieldwork phase delivered more detailed insights based on students’ lived experiences. For instance, although the literature emphasized general stressors like fear of infection and academic pressures, the fieldwork uncovered culturally specific factors unique to Iran, such as the influence of familial expectations and the use of religious coping mechanisms In Iranian culture, strong familial bonds and collective decision-making significantly influenced participants’ responses and coping strategies. Additionally, religion is deeply woven into daily life in Iran, influencing both coping mechanisms and perceptions of stress and its resolution. These variations indicate that psychological distress manifests differently across sociocultural contexts. Previous research by Alizadeh et al. similarly underscored the significance of cultural factors in understanding mental health outcomes during crises. Further exploration of these disparities could lead to actionable recommendations for culturally sensitive interventions [11, 54].

## Conclusion

This study highlights antecedents, characteristics, and consequences of psychological distress among nursing students during pandemics. By identifying these factors, the findings offer critical guidance for health policymakers and educational planners in addressing mental health challenges during future crises. The research emphasizes the importance of proactive approaches, such as identifying and mitigating the root causes of psychological distress, to reduce its prevalence in future pandemics. Furthermore, early intervention programs targeting both symptoms and contributing factors can play a crucial role in reducing the long-term negative effects of psychological distress.

In our study, the integration of findings from the qualitative and theoretical phases led to the following operational definition. psychological distress refers to emotional, cognitive, behavioral, and physical challenges arising from social, familial, and psychological factors. Depending on these factors, psychological distress may result in either positive or negative consequences. The findings also emphasize the importance of customized interventions that address both the psychological and educational needs of nursing students during pandemics. Building strong e-learning systems, implementing stress management programs, providing online mental health resources, and promoting religious coping strategies are essential measures to reduce distress. Additionally, integrating self-care practices into the nursing curriculum could help prevent long-term adverse effects and improve students’ overall well-being.

What distinguishes our study is its integration of cultural and contextual factors into the analysis of psychological distress. While existing frameworks often adopt a general approach, our research identifies culturally specific antecedents, such as reliance on religious coping mechanisms and the impact of familial expectations, particularly relevant in the Iranian context. Additionally, our findings emphasize the dual nature of psychological distress, demonstrating that while it can result in negative outcomes like academic setbacks, it also fosters resilience and post-traumatic growth (PTG). This nuanced understanding broadens the conventional definition by including positive transformations, an area less emphasized in prior frameworks.

Our hybrid concept analysis synthesizes theoretical insights with empirical data, offering a more comprehensive and practical definition. This approach builds upon existing definitions by:

### Contextualizing distress

Highlighting the interplay of academic, social, and health-related stressors specific to nursing students during pandemics.

### Cultural sensitivity

Incorporating sociocultural dynamics, which are often underrepresented in global frameworks.

### Broadening the scope

Introducing PTG as a dimension of psychological distress, illustrating how adverse experiences can lead to personal and professional growth.

These additions refine the concept of psychological distress and provide actionable insights for educational and mental health interventions tailored to nursing students in diverse contexts.

This study had several limitations. First, the term “psychological distress” carries a negative stigma, which may have influenced students to withhold accurate information during interviews. To mitigate this, the researcher made efforts to build trust with participants by guaranteeing the confidentiality of their responses. Social distancing measures also restricted access to students, and some were hesitant to participate in face-to-face interviews. As a result, telephone interviews were conducted instead. While this adjustment was necessary due to pandemic restrictions, it may have hindered the ability to observe non-verbal cues, which are often critical in qualitative research. Additionally, conducting interviews over the phone may have affected the depth of responses, particularly for participants who felt less at ease discussing sensitive topics in this format.

Another limitation is the absence of quantitative data, which limits the generalizability of the findings. Including quantitative measures could strengthen the study’s validity and provide more robust support for the qualitative results. Additionally, the findings are deeply embedded in the cultural context of Iran, where societal norms, values, and traditions significantly influence individual behaviors and perspectives. Consequently, demographic characteristics may reflect broader cultural dynamics, meaning the findings might not be directly transferable to other sociocultural contexts.

The absence of quantitative data further restricts the generalizability of the results. Future research should consider a mixed-methods approach to gain a more comprehensive understanding of psychological distress. Using quantitative measures, such as validated psychometric instruments, would enhance the credibility of the findings. Additionally, future studies should investigate the long-term impacts of psychological distress and evaluate the effectiveness of suggested interventions. This includes exploring ways to adapt cultural and educational systems to better support nursing students during global crises.

## Electronic supplementary material

Below is the link to the electronic supplementary material.

Supplementary Material 1

Supplementary Material 2

Supplementary Material 3

## Data Availability

The datasets used and/or analyzed during the current study are available from the corresponding author upon reasonable request.

## Abbreviations

WHO: World Health Organization
PRISMA: Preferred Reporting Items for Systematic Reviews and Meta-Analyses
Pubmed: Public/Publisher MEDLINE
MeSH: Medical Subject Headings
EMTREE: EMBASE Tree
MP3: MPEG Audio Layer III
COVID-19: Coronavirus Disease
