# Supplementary material for: Psychological distress among nursing students during the COVID-19 pandemic: a hybrid concept analysis
Source: BMC Psychol. 2025 Mar 8;13:218. doi: 10.1186/s40359-025-02562-x (PMC11889799; doi:10.1186/s40359-025-02562-x)
Supplement: Supplementary file 1 — Supplementary Material 1 [file 40359_2025_2562_MOESM1_ESM.docx]

**Table. 1**: Characteristics of the articles included in the study

|  | **Author and year** | **Study design** | **country**  **Sample/** | **Attributes** | **Consequences** | **Antecedents** |
| --- | --- | --- | --- | --- | --- | --- |
| **1** | **Bai et al(2021) (49)** | Cross-sectional | Beijing, China/983 nursing students | Irritability, Uncontrollable worry, Trouble relaxing, and Depressed mood |  | A specific phase of the current pandemic |
| **2** | **Alici et al(2020) (50)** | Descriptive correlation design | Turkey/234 nursing students | anxiety and fear |  | Gender , year in nursing school, place of residence, family or relatives infected with COVID‐19, home quarantine, and satisfaction with the remote learning |
| **3** | **Almhdawi et al (2021) (51)** | Cross-sectional study | Jordan/485 healthcare students at Jordanian universities | depression, anxiety,  and stress | negative impacts on their learning and wellbeing |  |
| 4 | **Grande et al (2021) (52)** | A cross‐sectional study | Saudi Arabia/484 Saudi NSs | Anxiety symptoms |  | Havingknown a COVID‐19 positive person, such as a family member or friend, |
| 5 | **Bahçecioğlu Turan et al (2021) (53)** | Descriptive and cross‐sectional. | Turkey/456 of nursing students | Anxiety |  | Gender, year of study, choosing the profession willingly, having anxiety for the profession |
| 6 | **Bai et al (2021) (54)** | Cross-sectional study | China/1,070 nursing students | Anxiety and depression |  | Students who chose nursing as their future career following the COVID-19 outbreak had less severe depression and anxiety compared to those who did not choose nursing |
| 7 | **Banstola et al (2021) (55)** | Descriptive cross-sectional study | Nepal/144 nursing students | Anxiety |  | Religion. |
| 8 | **Basheti et al (2021) (56)** | Descriptive cross-sectional study | Jordan/450 students studying a healthcare-oriented degree(medicine, dentistry, , and other) Pharm. D., pharmacy, nursing | Anxiety and depression |  | Smoking, lower family income and use of medications |
| 9 | **Bashir et al (2020) (57)** | Cross-sectional observational study | Pakistan/523 health science students | Anxiety and depression |  | Age, gender, education status, and COVID-19 positive patient, |
| 10 | **Begam, and Devi (2020) (58)** | Cross sectional descriptive | India/244 nursing students | Stress |  |  |
| 11 | **Beisland et al (2021) (59)** | Cross‑sectional study | Norway/2605 of nursing students | Fear | Quality of life | Level of education |
| 12 | **Brouwer et al (2021) (15)** | Correlational, design | USA/255 undergraduate or graduate nursing student. | Psychological distress |  | Self-identification, self-care practices |
| 13 | **Cici and Yilmazel (2020) (60)** | Descriptive study | Turkey/461 nursing students | Anxiety |  | Fear of being infected, mentally  affected due to pandemic |
| 14 | **Dalcalı et al (2021) (61)** | Descriptive and correlational type | Turkey/283 nursing students | anxiety | Reduce of sleep quality | feelings of fear |
| 15 | **Kumari Deo et al (2019) (62)** | Cross sectional descriptive study | Nepal/184 nursing students | Anxiety and depression | Insomnia | Reading and hearing news on increasing COVID -19 cases, long duty hour makes, spending a lot of money on recharge for mobile data for the online classes, age, marital status |
| 16 | **El-Noor (2021) (63)** | Cross-sectional design | Gaza/345 nursing students | Anxiety |  |  |
| 17 | **Emory et al (2021) (64)** | Cross-sectional design | United States/564 nursing students | Fear/anxiety |  |  |
| 18 | **Eweida et al(2020) (65)** | Cross-sectional descriptive study | Egypt/150 intern nursing  students | Felt under strain, worthless and depressed |  | Age, clinical experience and clinical placement |
| 19 | **Fitzgerald and Konrad (2020) (66)** | Descriptive study | USA/50 nursing students | Anxiety |  | Foster a structured learning environment; abide by the course schedule; communicate changes or updates in a timely fashion; adapt assignments to fit the learning environment; practice self‐care; and extend grace. |
| 20 | **Franzen et al (2021) (67)** | Cross-sectional | Switzerland/915 in eight different health-related tracks | Depression , Anxiety, stress | Lower academic satisfaction, well being | Gender, age |
| 21 | **Gao et al (2021) (68)** | Cross-sectional | China/1,780 college nursing students | Depression, anxiety, stress | PTSD, insomnia, | Gender, place of residence, education level |
| 22 | **Göl and Erkin (2021) (69)** | Cross‐sectional study | Turkey/2630 nursing students | Mental problems |  | Less sleep and diet |
| 23 | **García-González et al (2021) (70)** | Cohort cross-sectional | Spain/460 nursing students | Anxiety |  | Year of the degree program, being female,  The duration of the quarantine |
| 24 | **Gotlib et al (2021) (71)** | Cross-sectional, national online survey | Poland/790 Polish undergraduate nursing students |  |  | Lack of knowledge about the new type of coronavirus, income, routes of transmission, and the methods of COVID-19 diagnosis and treatment |
| 25 | **Hasanpour et al (2021) (72)** | Cross-sectional | Iran/174 nursing students |  |  | Having chronic  diseases, long time thinking about COVID-19  and death of family members, relatives or friends due to COVID-19 |
| 26 | **Hausike et al (2021) (73)** | Quantitative study that used an online survey | Japan/439 nurses and 340 current nursing students | Anxiety/fear | Decreased score of motivation, |  |
| 27 | **Huang et al (2020) (74)** | Comparative study | China/802 nursing college students | Anxiety, fear ,sadness  anger |  |  |
| 28 | **Kadappuran et al (2020) (75)** | NR | India/211 nursing students | Depression ,anxiety and stress, psychological distress | Disruption to their daily lives. | Women and urban people had more anxiety, family support, Government support, age, level of education |
| 29 | **Kim et al (2021) (76)** | A Cross-Sectional Survey | USA/173 nursing  students | Stress, anxiety,  and depression |  | Spiritual support, resilience, level of family functioning |
| 30 | **Kochuvilayil et al (2020) (77)** | Cross-sectional | Australia and India/99 Australian and 113 Indian undergraduate nurses | Anxiety | Difficulty leeping, concentrating and eating |  |
| 31 | **Labrague (2021) (78)** | Cross-sectional | 301 student nurses | Stress | Reduced life satisfaction and poorer psychological well-being. | Resilience |
| 32 | **Laranjeira et al (2021) (79)** | Cross-sectional | 1075 nursing students | Depressive  symptomatology |  | COVID-19 diagnosis in their household; their household income had been affected by the COVID-19 pandemic |
| 33 | **Li et al (2021) (80)** | Cross-sectional | China/348 home-quarantined nursing  students | High perceived stress, difficulty falling asleep or sleeping all night; consumed junk food in excess; neglected their appearance; felt headaches, stomach aches, and back pain; and lacked the patience or desire to exercise. depression and PTSS | Reduce life satisfaction | Male gender and insufficient social support were common risk factors for anxiety, |
| 34 | **Thomas (2022) (81)** | Used both quantitative and qualitative methods | USA/2326 undergraduate  students | Anxiety Depression PTSS |  |  |
| 35 | **Liu et al (2021) (82)** | Cross-sectional | China/1,070 nursing students | Stress |  | Male gender and being a senior nursing student economic loss during the COVID-19 pandemic |
| 36 | **Marcén-Román et al (2021) (83)** | Cross-sectional | Spain/252 university students | Psychological discomfort stress |  | Females  and last-year students, those who do not work and those who had come into less contact  with COVID-19. |
| 37 | **Miao et al (2021) (84)** | Cross-sectional | China/746 nursing students | Anxiety ,depression  stress | Emotion Regulation expression inhibition, |  |
| 38 | **Nania et al (2021) (85)** | NR | Italy/934 Italian university students | Depression, anxiety, and stress psychological distress |  |  |
| 39 | **Franzen et al (2021) (67)** | Cross-sectional | Switzerland./2835 | Anxiety and stress depression | Psychological well-being | Being female, Academic satisfaction |
| 40 | **Oducado(2021) (86)** | Cross-sectional | Philippines/175 nursing students | Anxiety |  | Lower satisfaction gender |
| 41 | **Pourghaznein et al (2021) (87)** | Cross-sectional | Iran/607 Iranian medical students |  |  | Gender, degree level, personal infectious status, family infectious presence and marital status |
| 42 | **Rafael et al (2021) (88)** | Cross-sectional | Brazil/477 students and workers |  |  | Fear of COVID-19, associated with diagnosis and belonging to groups at risk, perceptions about the family atmosphere, violence and the feeling of loneliness |
| 43 | **Rasmussen et al (2022) (89)** | Cross-sectional | Australia/Australia 2907 undergraduate  nursing and/or midwifery students | Psychological distress |  | Availability of personal protective equipment |
| 44 | **Rymer-Diez et al(2021) (90)** | Cross-sectional | Spain/122 |  |  | Psychosocial impact of the pandemic, adjustment to new modes of teaching and learning, and concerns about course progression and career |
| 45 | **Lampasona, et al (2021) (91)** | Cross-sectional | Italy/525 Nursing Students | Stress, depressive symptomatology, anxiety and distress |  | Female gender" "Low economic status", "Low health status" and "To be a smoker" |
| 46 | **Şentürk and Bakır(2021) (92)** | Descriptive and cross-sectional design. | Turkey/584 Nursing Student | Anxiety | Psychological  wellbeing | Age  Gender  Income  academic achievement |
| 47 | **Kaplan Serin (2021) (93)** | Descriptive and cross-sectional | Turkey/1107 and 344 of students | Depression, anxiety, stress |  | Sleep and eating patterns  gender  chronic diseases  economic conditions |
| 48 | **Ahmed et al (2021) (94)** | Descriptive cross-sectional | Pakistan/374 students | . |  | Financial issues, |
| 49 | **Sheroun et al (2020) (95)** | Cross-sectional | India/427 Nursing Students |  |  | Gender, grade |
| 50 | **Simionescu et al (2021) (96)** | NR | Romania/526 nursing students | Perceived stress |  | Gender and environment |
| 51 | **Kim et al (2020) (76)** | Cross-sectional | USA/173 students | . |  | Levels of resilience and family functioning, spiritual support  being employed students |
| 52 | **Sun et al (2020) (97)** | Cross-sectional | China/474 Students, Nursing |  |  | Male, sophomores and infrequent use of prevention measures  Innate resilience factors, medical history, insomnia,. |
| 53 | **Tanji and kodoma(2021) (98)** | Cross-sectional | Japan/289 nursing students |  |  | Medical history, concerns in college life (keeping up with classes, getting a job and proceeding to the next level of education, and friendship in college), subjective economic status, sense of fulfillment in college life, and insomnia |
| 54 | **Turan et al (2021) (53)** | Descriptive and cross‐sectional. | Turkey/456 nursing student | Psychological distress | Lacking the will to fight the difficulties encountered, to having low job satisfaction, and to perceiving their working environment negatively decreases the attitudes toward the nursing profession | Females, third‐year students, and students who willingly chose the nursing profession |
| 55 | **Uğurlu et al (2020) (99)** | NR | Turkey/411 nursing students | Anxiety |  | Age, gender, stayed with their family, number of people in the household  place of living  Being overweight |
| 56 | **Revertte-Villarroya et al (2021) (100)** | Transversal descriptive study | Spain/305 nursing students | Depressive  symptoms anxiety symptoms stress symptoms |  | Age, access to university, average marks, mental well-being self-esteem, emotional exhaustion, and sense of coherence |
| 57 | **Vitale et al (2020) (101)** | An observational descriptive study | Italy/285 Nursing students |  | General health of the body | Gender |
| 58 | **Yazici et al (2021) (102)** | Descriptive and cross-sectional study | 124 Nursing students |  |  | Fear of infecting family members and the feeling of uncertainty |
| 59 | **Yüksekol et al (2021) (103)** | Cross-sectional | Turkey/131 Nursing students | Fear of COVID-19 Anxiety |  | Smoking  financial problems that may occur in the future, the possibility of contracting COVID-19 and losing loved ones |
| 60 | **Zukhra et al (2021) (104)** | Web-based cross-sectional survey | Indonesia/247 Nursing students |  |  | Healthy lifestyle behaviors, staying at home and doing activities together with family and doing positive activities  age |
