# Supplementary material for: Psychological distress among nursing students during the COVID-19 pandemic: a hybrid concept analysis
Source: BMC Psychol. 2025 Mar 8;13:218. doi: 10.1186/s40359-025-02562-x (PMC11889799; doi:10.1186/s40359-025-02562-x)
Supplement: Supplementary file 2 — Supplementary Material 2 [file 40359_2025_2562_MOESM2_ESM.docx]

**Table 3.** The Participants’ Demographic Characteristics

| **Place of living**  **Dormitory/personal residence** | **Academic semester** | **Age** | **Participant code** |
| --- | --- | --- | --- |
| Dormitory | Bachelor.3 | 19-23 | 1 |
| Dormitory | Bachelor2 |  | 2 |
| Dormitory | Bachelor4 |  | 3 |
| Dormitory | Bachelor4 |  | 4 |
| Personal | Bachelor4 |  | 5 |
| Personal | Bachelor5 |  | 6 |
| Personal | Bachelor5 |  | 7 |
| Dormitory | Bachelor5 |  | 8 |
| Dormitory | Bachelor6 |  | 9 |
| Dormitory | Bachelor6 |  | 10 |
| Dormitory | Bachelor6 |  | 11 |
| Dormitory | Bachelor6 |  | 12 |
| Dormitory | Bachelor6 |  | 13 |
| Dormitory | Bachelor2 |  | 14 |
| Dormitory | Bachelor3 |  | 15 |
| Dormitory | Bachelor4 |  | 16 |
| Dormitory | Bachelor5 |  | 17 |
| Dormitory | Bachelor6 |  | 18 |
| Dormitory | Bachelor7 |  | 19 |
| Dormitory | Bachelor7 |  | 20 |
| Dormitory | Bachelor6 |  | 21 |
| Dormitory | Bachelor6 |  | 22 |
| Dormitory | Bachelor3 |  | 23 |
| Personal | Bachelor3 |  | 24 |
