# Supplementary material for: Psychological distress among nursing students during the COVID-19 pandemic: a hybrid concept analysis
Source: BMC Psychol. 2025 Mar 8;13:218. doi: 10.1186/s40359-025-02562-x (PMC11889799; doi:10.1186/s40359-025-02562-x)
Supplement: Supplementary file 3 — Supplementary Material 3 [file 40359_2025_2562_MOESM3_ESM.docx]

Appendix 1.Interview Schedule And Protocol

| Interview no. | Date of interview | Interview start time | Interview end time |
| --- | --- | --- | --- |
| 1 | 2020-10-4 | 09:30 | 09:50 |
| 2 | 2020-10-18 | 10:40 | 11:00 |
| 3 | 2020-10-18 | 11:00 | 11:30 |
| 4 | 2020-10-18 | 11:30 | 11:50 |
| 5 | 2020-10-24 | 09:30 | 10:00 |
| 6 | 2020-10-24 | 10:00 | 10:20 |
| 7 | 2020-12-1 | 08:20 | 08:40 |
| 8 | 2020-12-1 | 09:00 | 09:30 |
| 9 | 2020-12-4 | 12:00 | 12:15 |
| 10 | 2021-1-8 | 11:00 | 11:20 |
| 11 | 2021-1-22 | 10:30 | 10:45 |
| 12 | 2021-1-29 | 09:00 | 09:20 |
| 13 | 2021-3-17 | 08:00 | 08:20 |
| 14 | 2021-3-17 | 09:00 | 09:30 |
| 15 | 2021-5-9 | 10:00 | 10:30 |
| 16 | 2021-5-9 | 11:00 | 11:20 |
| 17 | 2021-6-3 | 10:30 | 11:00 |
| 18 | 2021-6-3 | 11:00 | 11:20 |
| 19 | 2021-8-23 | 10:30 | 11:00 |
| 20 | 2021-10-10 | 08:45 | 09:15 |
| 21 | 2021-11-29 | 09:00 | 09:20 |
| 22 | 2022-1-18 | 10:30 | 10:50 |
| 23 | 2022-1-18 | 11:00 | 11:20 |
| 24 | 2022-2-20 | 10:00 | 10:20 |
